# Effects of Cognitive Bias Modification Training via Smartphones

**DOI:** 10.3389/fpsyg.2017.01370

**Published:** 2017-08-14

**Authors:** Ranming Yang, Lixia Cui, Feng Li, Jing Xiao, Qin Zhang, Tian P. S. Oei

**Affiliations:** ^1^Beijing Key Laboratory of Learning and Cognition and Department of Psychology, Capital Normal University Beijing, China; ^2^Criminal Justice College, China University of Political Science and Law Beijing, China; ^3^School of Psychology and CBT Unit, Toowong Private Hospital, University of Queensland Brisbane, QLD, Australia; ^4^Psychology Section, James Cook University Singapore, Singapore

**Keywords:** cognitive bias modification, attention, interpretation, smartphones, social anxiety

## Abstract

**Background and Objectives:** Negative cognitive biases have been linked to anxiety and mood problems. Accumulated data from laboratory studies show that positive and negative interpretation styles with accompanying changes in mood can be induced through cognitive bias modification (CBM) paradigms. Despite the therapeutic potential of positive training effects, few studies have explored training paradigms administered via smartphones. The current study aimed to compare the effectiveness of three different types of training programmes (cognitive bias modification-attention, CBM-A; cognitive bias modification-interpretation, CBM-I; attention and interpretation modification, AIM) administered via smart-phones by using a control condition (CC).

**Methods:** *Seventy-six* undergraduate participants with high social anxiety (Liebowitz Social Anxiety Scale, LSAS ≥ 30) were randomly assigned to four groups: CBM-A (*n* = 20), CBM-I (*n* = 20), AIM (*n* = 16), and CC (*n* = 20).

**Results:** The results showed that the effects of CBM training, CBM-I training, or AIM training vs. CC for attention yielded no significant differences in dot-probe attention bias scores. The CBM-I group showed significantly less threat interpretation and more benign interpretation than the CC group on interpretation bias scores.

**Conclusions:** The present results supported the feasibility of delivering CBM-I via smartphones, but the effectiveness of CBM-A and AIM training via smartphones was limited.

## Introduction

Research has shown that individuals who are extremely anxious tend to preferentially orient their attention toward threats, which influences their sensitivity to the environment (Fox and Beevers, 2016), and to interpret ambiguous information as threatening, which could reflect the down-stream effects of anxiety (White et al., 2016). While several psychotherapeutic procedures have been used to treat these biases (Tobon et al., 2011), recently, an increasing amount of research has focused on the use of Cognitive Bias Modification (CBM) procedures (Mackintosh et al., 2006; Wilson et al., 2006).

Researchers have developed experimental paradigms to modify cognitive biases, such as Cognitive Bias Modification-Attention (CBM-A) and Cognitive Bias Modification-Interpretation (CBM-I). Recent evidence has shown that CBM-A can reduce threatening attention bias (Kuckertz and Amir, 2015) and that CBM-I can promote an optimism interpretive bias among anxious individuals (Rozenman et al., 2014; Clifton et al., 2016). Furthermore, these modifications were accompanied by a decline in the level of anxiety (Beard et al., 2011; Brosan et al., 2011). In addition, the positive outcomes of both CBM-A and CBM-I were replicated in people with anxiety problems (Rozenman et al., 2014; Clifton et al., 2016) and anxiety disorders (such as Social Phobia, Amir et al., 2009; Ogniewicz et al., 2014). Other studies (Timpano et al., 2009; Amir and Taylor, 2012) showed that people with anxiety also benefited from multi-session bias modification at a 4-month follow-up. Meta-analyses have examined the efficiency of CBM-A for anxiety and found an effect size of 0.61 (Hakamata et al., 2010). Another meta-analysis showed that CBM had a medium effect on biases (*g* = 0.49) and that the effect of CBM-I was stronger (*g* = 0.81) than that of CBM-A (*g* = 0.29) (Hallion and Ruscio, 2011). Beard et al. (2011) found that the attention and interpretation modification (AIM) treatment produced medium to large effects on social anxiety. A problem with using CBM alone or in combination was that participants reported that the procedures were tedious and repetitive. As a result, participants became tired and impatient (Beard et al., 2011). This could impair training adherence and lead to a high dropout rate.

With the increasing number of people who own a smartphone, interventions that incorporate smartphones have become increasingly popular in recent years (Garritty and El Emam, 2006). Carlbring et al. (2012) delivered CBM-A training via the internet, and the post treatment and 4-month follow-up results revealed a significant time effect on all measured dimensions (social anxiety scales, general anxiety, and depression levels, quality of life). Enock et al. (2014) demonstrated the feasibility of delivering CBM-A via smartphones in short, frequent sessions as well as the feasibility of conducting a relatively large, low cost, minimal contact, web-based RCT. Additionally, internet-based CBM-I had superior effects on interpretations (Salemink et al., 2014). The use of online CBM-I with anxious youth holds promise as an effective and easily administered component of treatment for child social anxiety (Reuland and Teachman, 2014). CBM training can be promoted along with the increasing number of medical-related applications available for smartphones. Moreover, as specialized mobile phones contain additional computing capabilities, they are likely to be the principal platforms for the development of the next generation of clinical applications (Boulos et al., 2011). Training in diverse locations could foster the generalization of clinical benefits. In addition to being popular and mobile, smartphone interventions also provide high accessibility for individuals seeking help and could reduce the cost of treatment (Kazdin and Rabbitt, 2013). The last advantage of delivering CBM through smartphones is that the approach could augment CBM's training effects. A high dose and frequency of training is a prerequisite for creating enduring changes in cognitive habits. Frequent training is easy with smartphones as participants can undergo training anywhere throughout the day.

In general, CBM training research is moving toward delivering longer interventions in naturalistic settings (MacLeod et al., 2009), but few CBM intervention studies have specifically targeted smartphones, and little is known about the effects of different CBM training programmes provided via smartphones. This is particularly true in China, where the penetration of smartphones is now widespread. With the advancement of modernization and economic progress, the rate of anxiety problems and anxiety disorders in China is increasing (Guo, 2000), yet treatment for anxiety problems is still not as well developed as in the Western world. The use of smartphones for the treatment of anxiety and anxiety-related problems needs to be tested in China. The Western literature reviewed above suggests that combined CBM is likely to be more effective than each programme alone. Thus, in this study, we hypothesized that AIM would produce better outcomes than either CBM-A or CBM-I alone and that these three conditions would have better outcomes than the control condition.

## Methods

### Participants

One hundred four participants with scores above 30 on the Liebowitz Social Anxiety Scale (LSAS; Liebowitz, 1987) were selected from 490 undergraduate or graduate students at three different universities to participate in the study. In a pre-workshop evaluation conducted before the programme, we excluded those who scored 48 or higher on the LSAS and were likely to suffer from anxiety disorders or depression. Every participant read and signed an informed consent form. Our final sample included 76 Chinese undergraduate and graduate students (26 men and 50 women) with average age of 21.23 years old (*SD* = 2.42). The mean trait anxiety score for this group was 38.11 (*SD* = 5.12). This study was approved by the Institutional Review Board of Capital Normal University. A power analysis with the parameters 0.80, *p* < 0.05 and a medium effect size showed that the total sample size was 72 for an *F*-test among the factors of a 4 × 2 repeated measures ANOVA; thus, our sample of 76 participants was adequate.

### Assessment materials

#### Attention bias modification assessment task

**Figure 1:**
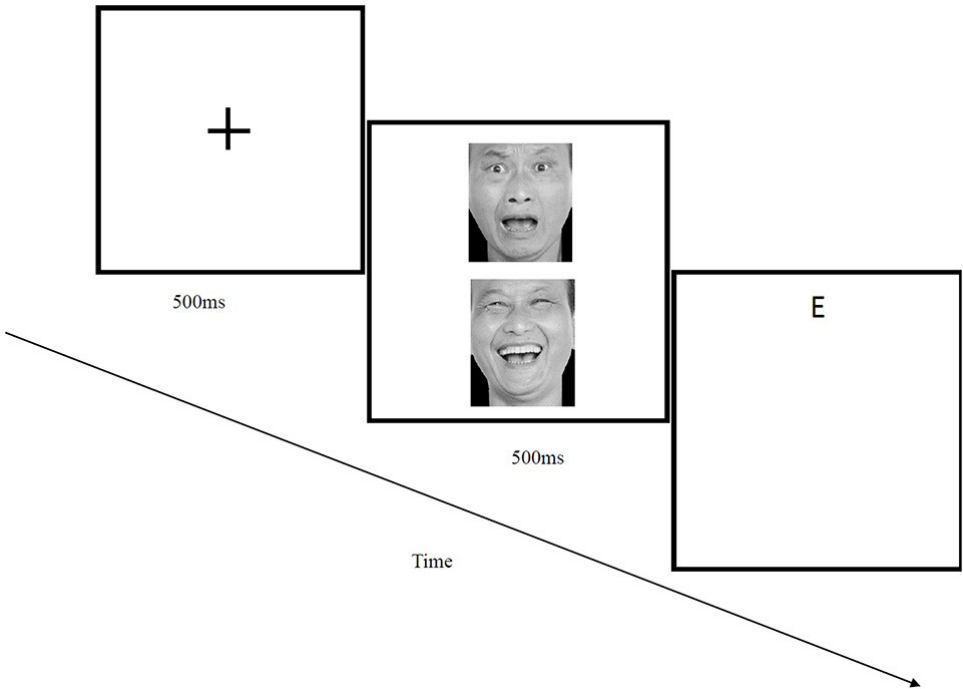
Example of a single trial for the dot-probe attention bias assessment.

The *Attention Bias Modification Assessment* Task consisted of a dot-probe task similar to that described in Macleod et al. (1986). In this paradigm, a fixation cross appeared for 500 ms, followed by the simultaneous presentation of two faces for 500 ms. One face appeared at the top of the screen, and the other appeared at the bottom. Immediately thereafter, a probe replaced one of the two faces. The probe was either an E or an F. The participants' task was to press one of two buttons on the screen as quickly as possible to indicate whether an E or F was presented. If the subject did not respond after 2,000 ms, the program automatically moved on to the next trial. The top and bottom positions of the probe letters and faces were randomly assigned, and the stimuli included 8 pairs of neutral/negative pictures, 8 neutral/neutral pictures and 8 neutral/neutral pictures (with no letters present). Each face was presented on the screen at the top and at the bottom four times. Of the total trials, 128 (2/3 of the total number) included 8 neutral faces and 8 negatives, with the probes appearing at an equal frequency in the position of negative face and neutral face [2 (negative face position: top or bottom) × 2 (probe type: E or F) × 4 (repetition)]; 32 trials (1/6 of the total number) included 8 neutral faces and 8 neutral faces, with the probes appearing at an equal frequency in each position of the neutral faces [2 (neutral face position: top or bottom) × 2 (probe type: E or F)]; and the remaining 32 trials (1/6 of the total number) were clueless trials (8 neutral face pairs × 2 (neutral face position: top or bottom) × 2 (probe type, E or F). Every test took ~5 min. All the face stimuli were from the Chinese Facial Affective Picture System (CFAPS; Wang and Luo, 2005) and were balanced across gender (male vs. female; See Figure 1). The program recorded automatically the participants' reaction time during the test.

#### Interpretation bias assessment

**Figure 2:**
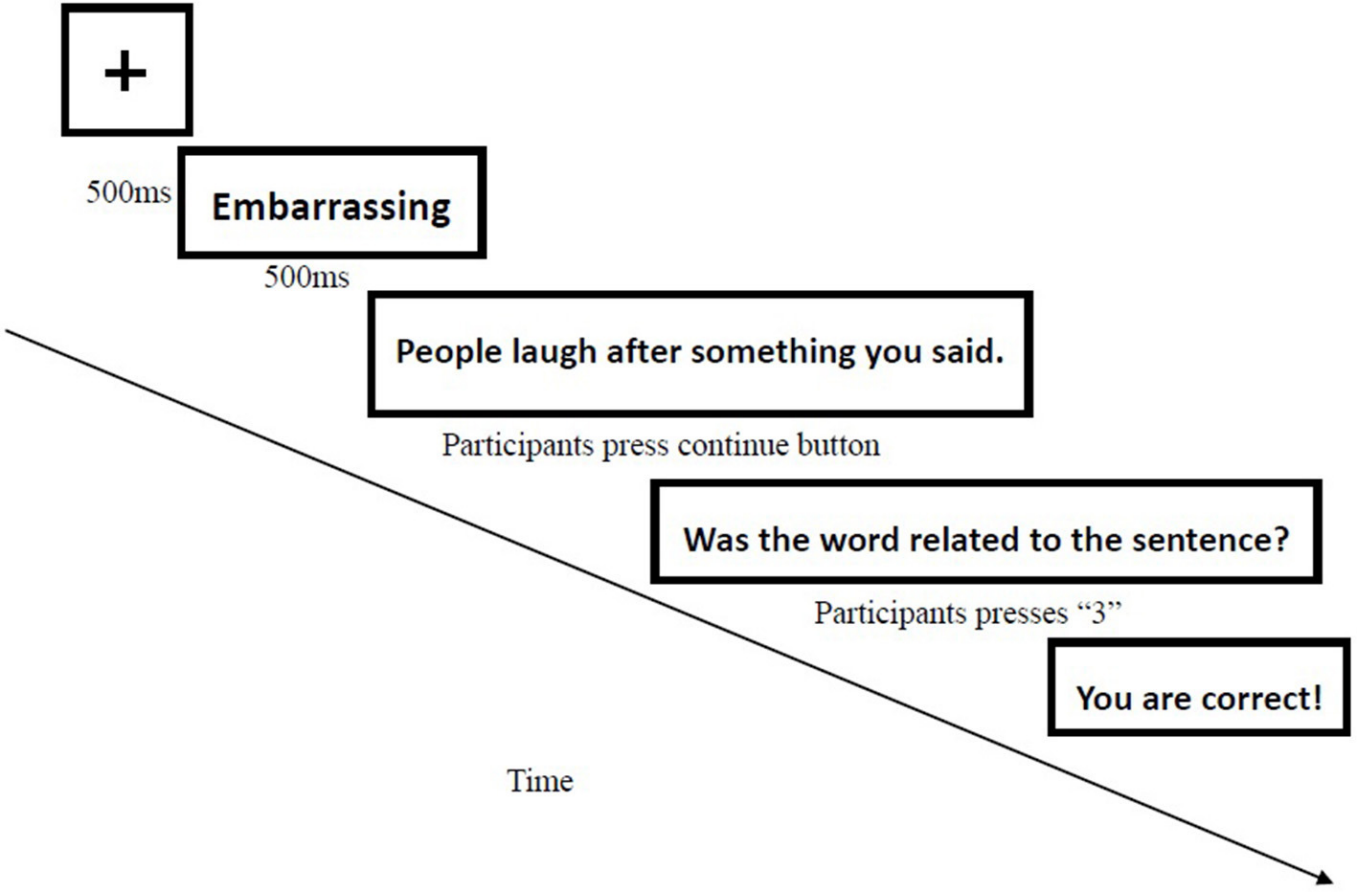
Example of a single trial in the WSAP interpretation bias assessment.

The CBM-I assessment procedure was identical to the Word Sentence Association Paradigm (WSAP; Beard and Amir, 2009). Each WSAP trial comprised four phases (See Figure 2). The participants completed 40 trials related to ambiguous social situations. The next trial began immediately after the participants responded. CBM-I ambiguous scenarios were based on those used by Mathews and Mackintosh (2000) and were revised to reflect Chinese students' situations. With reference to the 24 types of social contact situations described in the Liebowitz scale, we translated, rewrote and classified the situations into 20 types of social contact situations in accordance with the national context of China.

#### Liebowitz social anxiety scale (LSAS; Liebowitz, 1987)

The LSAS consists of 24 social situations (e.g., giving a speech) that socially anxious individuals commonly fear. For each situation, the participants rated their fear (0–3) and avoidance (0–3). The Chinese version of the LSAS has good reliability and validity (He and Zhang, 2004).

#### State trait anxiety (STAI; Spielberger et al., 1983)

The State Trait Anxiety Inventory includes two parts with 20 items each. The first part is the STAI-S, which is used to assess temporary unpleasant emotional experiences, such as tension, fear, worry and feelings of anxiety. The second part is the STAI-T, which is used to assess the tendency toward anxiety in an individual's relatively stable personality. The internal consistency reliability (Cronbach's α) of the Chinese versions of the STAI is 0.88, and the norm score for trait anxiety among Chinese undergraduates is 43.31 (*SD* = 9.20) (Li and Qian, 1995).

### Procedure

**Figure 3:**
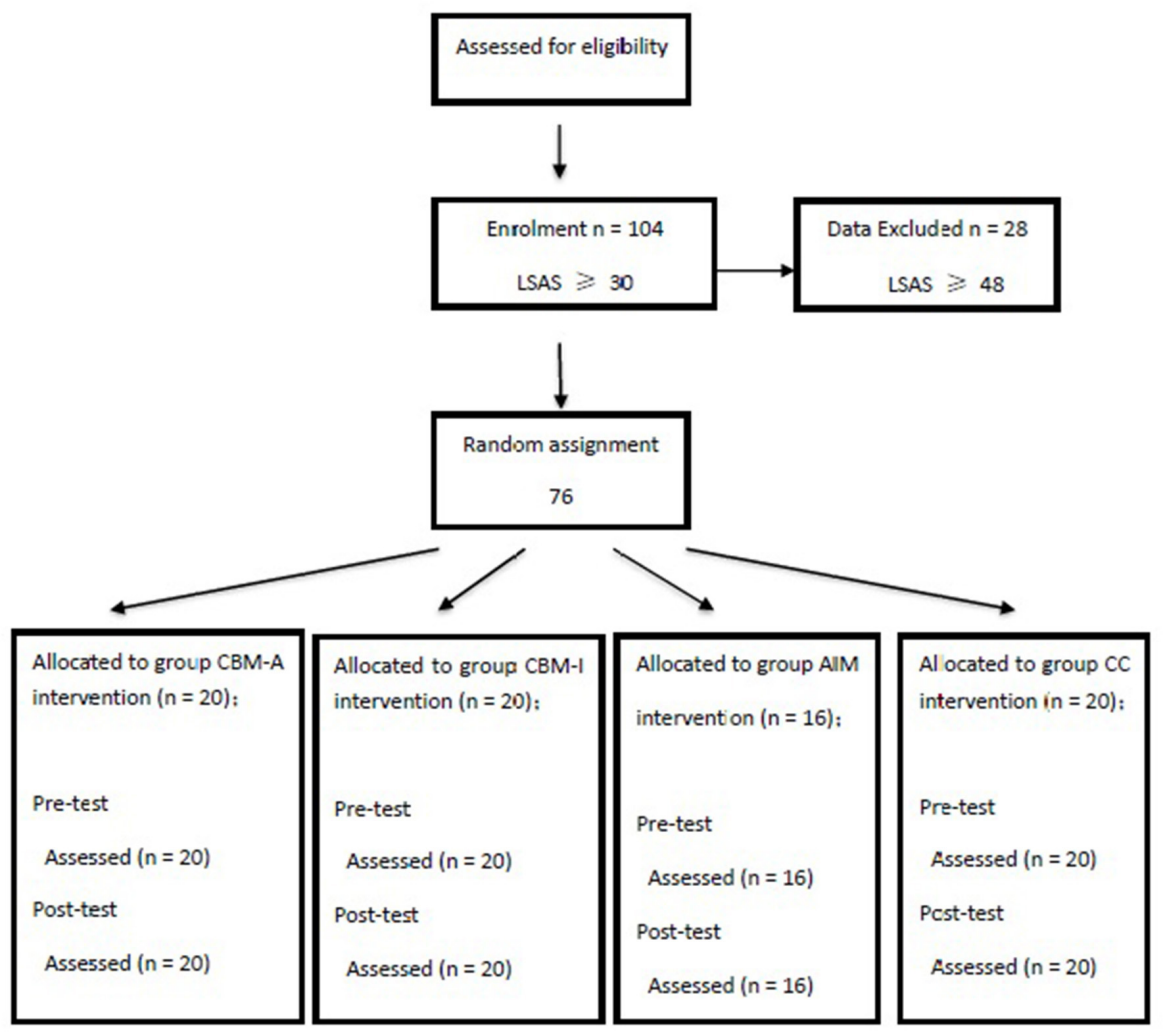
Flow chat of participants through each stage of the study.

See the flow of participants through each stage of the study in Figure 3. The participants completed the written informed consent form at the beginning of the pre-assessment. The consent form stated the purpose of this study: to evaluate the usefulness of smartphone based treatments for anxiety. However, it did not provide information regarding the rationale for either of the two conditions. The consent form also stated that the participants would be randomly assigned to one of the four groups: one receiving the cognitive bias modification-attention training, one receiving the cognitive bias modification-interpretation training, one receiving the attention and interpretation modification training, and the other group experiencing control conditions. The participants completed baseline assessments that included the STAI self-report measure, an attention bias assessment and an interpretation bias assessment. The pre-assessment took approximately 10 min. Following the pre-assessment, the participants were randomly assigned to one of the four conditions: CBM-A or CBM-I or AIM or CC. Eligible individuals visited an inter-linkage that outlined the training protocol and received instructions for accessing the training web page on their handheld devices.

#### CBM-A training

The CBM-A training was similar to the attention bias assessment. The difference was that the probe (either the letter “E” or “F”) only appeared at the location of the positive face. The stimuli used for the attention bias modification (ABM) task (dot probe task) were 40 positive-negative face pairs (i.e., happy and angry) and 40 neutral-neutral face pairs. During the CBM-A training, the participants completed 800 trials that comprised various combinations of probe type (E or F), probe position (top or bottom), and face type (positive or negative). Six hundred forty out of the 800 trials (i.e., 80% of the trials) included 40 positive faces and 40 negative faces, and the probe always replaced the positive face [2 (negative face position: top or bottom) × 2 (probe type: E or F) × 4 (repetition)]. Although, nothing specific to direct attention away from negative faces was provided, in 80% of the trials, the position of the negative face predicted the position of the probe (i.e., at the location opposite the disgust face). The remaining 160 trials (i.e., 20% of the trials) included 40 neutral-neutral face pairs [2 (neutral face position: top or bottom) × 2 (probe type: E or F)]. The training phrase lasted 40 min.

#### CBM-I training

The CBM-I training was similar to the WSAP except that the participants received feedback on their responses. Specifically, after the participants responded regarding the relatedness of the word and the sentence presented, the smartphones provided feedback regarding their response. The participants received positive feedback when they endorsed benign interpretations or rejected threat interpretations in the ambiguous sentences, and they received negative feedback when they endorsed threat interpretations or rejected benign interpretations. This feedback aimed to strengthen a benign interpretation bias and eliminate the threat interpretation bias. The participants completed 160 training trials. The CBM-I session lasted approximately 40 min.

#### AIM training

Participants in the AIM group received the same smartphone tasks as those in the CBM-A and CBM-I group. However, they only completed half the tasks for CBM-A and CBM-I. First, the participants received 400 CBM-A training trials. Of these trials, 320 (i.e., 80%) included 20 positive faces and 20 negative faces. The remaining 80 trials (i.e., 20%) included 20 neutral face pairs. The training phrase lasted 20 min. Then, the participants completed CBM-I training, which included 80 training trials. The CBM-I session lasted approximately 20 min. The entire AIM session lasted approximately 40 min.

#### CC group

The CC group received the same smartphone tasks as the AIM group, but the process was different. In the probe task, when negative faces were presented to the CC group (i.e., positive-negative trials), the probe appeared with equal frequency in the position of the negative face and positive face. Therefore, 160 out of 400 trials (i.e., 40%) were positive-negative, with the probe following the negative face. The remaining 80 trials (i.e., 20%) included only neutral faces, as in the CBM-A. Neither the disgust face nor the neutral face had signal value regarding the location of the probe. In the interpretation task, the CC group's task was identical to that of the CBM-I except that the feedback contingency was lowered to 50%. Specifically, the participants received positive feedback when they made threat interpretations on half of the trials and negative feedback when they made threat interpretations for the remaining half of the trials. This contingency was the same for benign interpretations. Thus, the control group was reinforced equally for making threat and benign interpretations. The CC was not intended to change the participants' interpretation significantly in either direction. The CC session lasted approximately 40 min.

After the CBM-A, CBM-I, AIM or CC training, the participants completed a post-assessment that was similar to the pre-assessment. All the participants were compensated at the rate of ¥40/h.

## Results

### Preliminary analyses

Descriptive statistics, including means and standard deviations (SD) for all outcome scores, are presented in Table 1. The participants assigned to the four groups did not differ in terms of demographic characteristics or baseline outcomes.

**Table 1:** The means and SDs of the four groups over time.

	**CBM-A *N* = 20**		**CBM-I *N* = 20**		**AIM *N* = 16**		**CC *N* = 20**	
	***M***	***SD***	***M***	***SD***	***M***	***SD***	***M***	***SD***
**SAI**								
Pre-test	36.55	7.92	41.70	9.18	37.44	8.29	39.50	8.26
Post-test	35.70	10.14	40.00	9.39	37.81	8.69	45.25	11.72
**ATTENTION BIAS**								
Pre-test	1.28	24.70	1.11	22.33	1.25	31.53	1.19	31.02
Post-test	1.17	20.01	1.02	20.17	1.22	27.71	1.03	39.07
**BENIGN INTERPRETATION**								
Pre-test	−92.73	113.34	−102.73	145.54	−96.61	109.56	−95.15	103.65
Post-test	−117.22	140.21	−214.21	120.43	−116.56	118.64	−90.81	136.34
**NEGATIVE INTERPRETATION**								
Pre-test	271.22	204.10	269.27	190.23	246.06	211.67	256.38	321.53
Post-test	222.12	189.60	106.75	178.08	197.95	167.23	222.57	351.15

*CBM-A, cognitive bias modification-attention; CBM-I, cognitive bias modification-interpretation; AIM, attention and interpretation modification; CC, control group*.

### State anxiety scores

To examine whether the training was influenced by anxiety state, a 4 × 2 mixed-design ANOVA was performed on the state anxiety data, with group (CBM-A, CBM-I, AIM vs. CC) as between subjects and time (pre vs. post-tests) as the within-subjects factors. The results showed neither group nor Time main effects were significant (*p* > 0.05). The Group × Time interaction effect was also not significant (*p* > 0.05). The results indicated that the differences between the groups and the data before and after the training were not influenced by anxiety state.

### Attention bias

The mean attention bias scores for the four groups were presented in Table 1. The attention bias was the time subtracting the mean response time for neutral-location probes from the mean response for threat-location probes in negative-neutral trials. Higher scores suggested that there were more biases toward negative faces. The mixed 4 × 2 ANOVA with Group and Time factors yielded no significant main effects for the time [*F*_(1, 72)_ = 6.01, *p* > 0.05, η^2^ = 0.04, *1*-β = 0.72] and for the group [*F*_(3, 72)_ = 7.15, *p* > 0.05, η^2^ = 0.07, *1*-β = 0.70]. There was also not significant Time × Group interactions [*F*_(3, 72)_ = 6.09, *p* > 0.05, η^2^ = 0.05, *1*-β = 0.75].

### Interpretation bias

The participants' reaction time on the performance-based interpretation task was measured to examine how negative and benign interpretations were related to the ambiguous sentences. This measurement revealed four types of reaction times: (a) acceptance of negative interpretations, (b) rejection of negative interpretations, (c) acceptance of benign interpretations, and (d) rejection of benign interpretations. Negative and positive bias indices were calculated for the reaction time data: negative bias = reaction times (reject negative—accept negative) and benign bias = reaction times (accept benign—reject benign). Higher scores suggested that there were more biases toward threat and benign interpretations, respectively. The lower the positive bias score, the more positive the interpretation was.

The mean negative and positive bias scores for the four groups over time are shown in Table 1. The mixed 4 × 2 ANOVA with group (CBM-A, CBM-I, AIM vs. CC) as the among-subjects factor and time as the repeated measure over time yielded significant main effects over time for negative bias [*F*_(1, 72)_ = 11.07, *p* < 0.001, η^2^ = 0.13, *1*-β = 0.90] and for positive bias [*F*_(1, 72)_ = 10.01, *p* < 0.001, η^2^ = 0_._22, *1*-β = 0.92]. Significant group main effects were also found for negative bias [*F*_(3, 72)_ = 9.17, *p* < 0.001, η^2^ = 0.14, *1*-β = 0.91] and for positive bias [*F*_(3, 72)_ = 13.11, *p* < 0.001, η^2^ = 0_._18, *1*-β = 0.92]. Finally, significant Time × Group interactions were detected for negative bias [*F*_(3, 72)_ = 12.22, *p* < 0.001, η^2^ = 0_._17, *1*-β = 0.93] and for positive bias [*F*_(3, 72)_ = 12.12, *p* < 0.001, η^2^ = 0.10, *1*-β = 0.94]. For outcomes with significant Time × Group interactions, separate follow-up repeated-measures ANOVA models tested whether the groups differed significantly between pre- and post-test.

**Figure 4:**
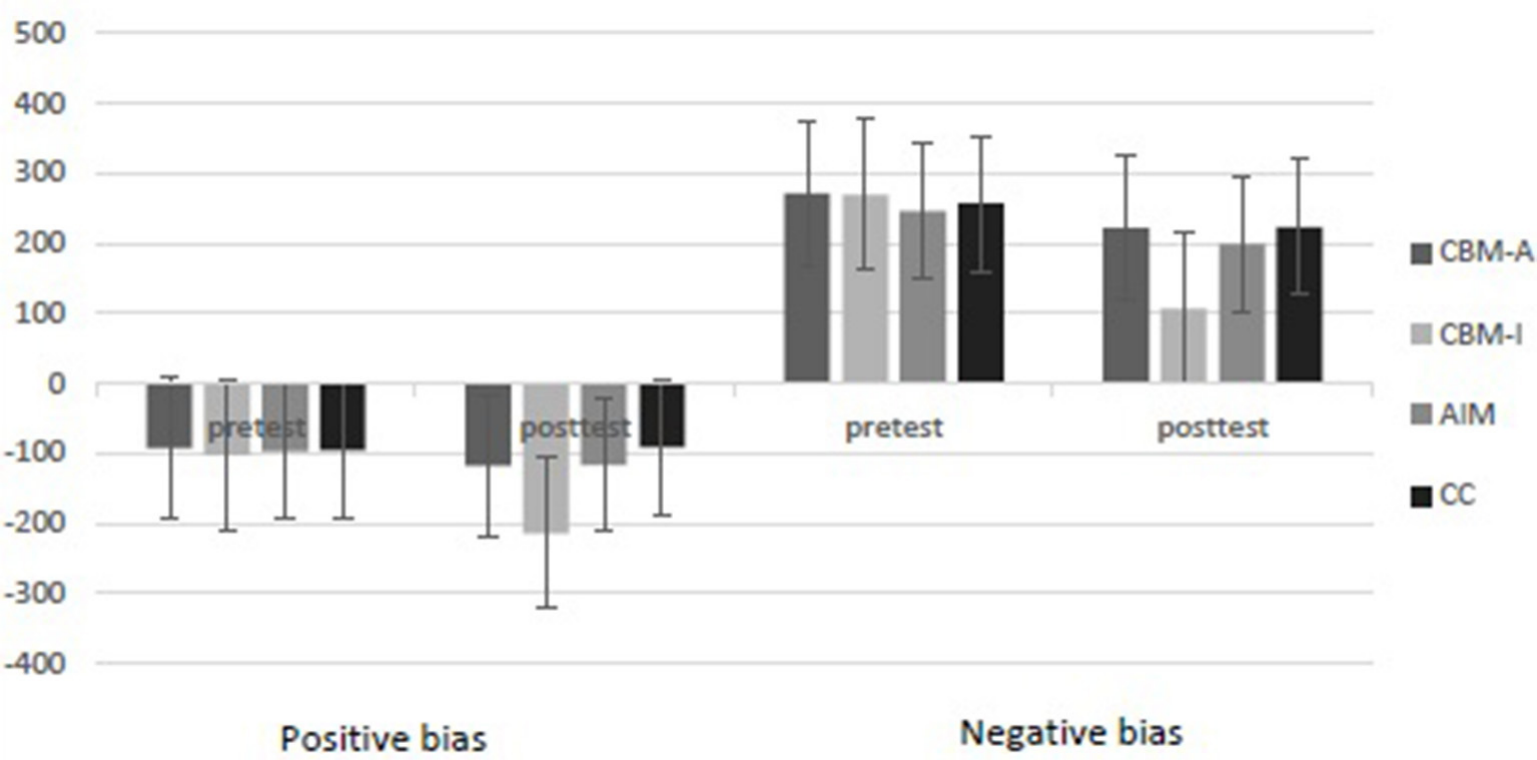
Comparison of the positive bias and negative bias scores among the four groups at pre-test and post-test assessments.

The mean positive bias scores for the four groups over time are shown in Figure 4. *Post-hoc* comparisons were conducted at the pre- and post-test. The results showed that there was no significant difference among the four groups at pre-test. At post-test, the positive bias scores in the CBM-I group were more negative and significantly higher than those in the CBM-A, AIM and CC groups (*p* < 0.001, *d* ≥ 0.50, *1*-β ≥ 0.90), but there was no significant difference among the CBM-A, AIM, and CC groups (*p* > 0.05, *d* ≤ 0.20, *1*-β ≥ 0.80). *Post-hoc* comparisons between pre- and post-test were conducted within the four groups. The results showed that the positive bias scores at post-test were more negative and significantly higher than at pre-test (*p* < 0.001, *d* = 0.55, *1*-β = 0.92) for the CBM-I group, but in the other three groups, there was no significant difference between pre- and post-test (*p* > 0.05, *d* ≤ 0.20, *1*-β ≥ 0.80).

The mean negative bias scores for the four groups over time are shown in Figure 4. *Post-hoc* comparisons were conducted pre- and post-test. The results showed no significant difference among the four groups at pre-test. At post-test, the negative bias scores of the CBM-I group were significantly lower than those of the CBM-A, AIM and CC groups (*p* < 0.001, *d* ≥ 0.50, *1*-β ≥ 0.90). However, there was no significant difference among the CBM-A, AIM and CC groups (*p* > 0.05, *d* ≤ 0.20, *1*-β ≥ 0.90). *Post-hoc* comparisons between pre- and post-test were conducted within the four groups. The results showed that the negative bias score at post-test was significantly lower than that at pre-test (*p* < 0.001, *d* = 0.57, *1*-β = 0.93) in the CBM-I group, but in the other three groups, there was no significant difference between pre- and post-test (*p* > 0.05, *d* ≤ 0.20, *1*-β ≥ 0.80).

## Discussion

In this study, we tested the effects of one session of CBM-A training, CBM-I training, AIM training and CC on participants' attention and interpretation bias using a dot-probe task and the WSAP task delivered via smart-phone. The results showed that the effects of CBM training, CBM-I training, and AIM training vs. CC on attention yielded no significant differences in the dot-probe attention bias scores. The CBM-I group showed significantly less threat interpretation and more benign interpretation than did the CC group in the interpretation bias scores.

Why was there a change in interpretation bias with CBM-I but not with AIM, which contains the same training? A reason might be that the training trials did not reach the minimum requirements for the effect onset. The participants in the AIM group received the same smartphone task as the CBM-A and CBM-I participants. However, they only finished half the number of tasks that the CBM-A and CBM-I participants did. According to the current literature regarding the WSAP (Amir and Taylor, 2012), 110 trials each time was the minimum needed for effect onset. In the present study, the participants only finished 80 trials per session and may not have reached the minimum requirements for the effect onset.

In contrast to prior research reporting the effective modification of attention through dot-probe training in healthy samples (Hakamata et al., 2010; Hallion and Ruscio, 2011; Mogoase et al., 2014), we found no evidence that CBM-A or AIM provided via smartphones can modify attention biases via single-session training. This result agreed with recent studies that did not successfully modify attention bias through ABM programmes (e.g., Carlbring et al., 2012; Boettcher et al., 2013; Rapee et al., 2013). Furthermore, home-based training encouraged participants to focus their attention more closely, which facilitated the learning of the rule (i.e., that the probe was more likely to occur in the location opposite the threat cues); consequently, changes in performance may not necessarily indicate changes in the attention bias toward threat (Mogg et al., 2017). However, we think that these failures do not negate the theoretical and empirical basis of ABM. Effective changes in bias change depend on intensive empirical study of the precise task conditions and modes of delivery.

In our study, one possible reason for the failure to change attention bias was the participants' engagement level. In interviews conducted after the experiment, the participants told us that they could not understand how the ABM programme would help them to reduce anxiety and easily becoming upset. In contrast, they reported they could understand the interpretation modification programme well and that CBM-I training felt more fun and more engaging than CBM-A training.

Another possible reason was that the level of pre-training attention bias among the present participants was low, which led to no significant change in attention bias. The participants' pre-training attention bias score moderated the efficacy of CBM–A (Amir et al., 2011). In this study, the participants were normal and only had high social anxiety; the ABM procedures might exert a greater impact on participants with an existing attention bias toward threat, as predicted with clinical models.

This study has several limitations. First, the participants' engagement level in CBM-A training was low. Second, the training trials did not reach the minimum requirements for the effect onset. Third, the sample size was rather low. Although, we had sufficient power to detect the influences and effect sizes described in the results section, future studies with larger sample sizes are needed to support the conclusion presented in the present study. In future studies, we can improve our experiment in the following ways: First, we will try to combine fun elements with training via smartphones to improve the participants' engagement level in ABM. Second, we need to increase the number of training trials to reach the minimum requirements for the effect onset. Third, we will increase the number of subjects and improve the effect sizes.

This was only a preliminary study. The failures to consistently modify biased attention to threat highlights the need for more reliable means of achieving change in the target cognitive process. The present results only initially showed the feasibility of delivering CBM-I via smartphones, but the effectiveness of CBM-A and AIM training via smartphones was limited. Before longer interventions within naturalistic settings are conducted, some research on CBM interventions specifically delivered through smartphones is necessary.

## Ethics statement

All procedures performed in studies involving human participants were in accordance with the ethical standards of the institutional and/or national research committee and with the 1964 Helsinki declaration and its later amendments or comparable ethical standards. Informed consent was obtained from all the individual participants included in the study.

## Author contributions

RY and LC the main writers and contributors of the paper and contributed equally to this work. LC the director of the research. FL took charge of the writing. JX revised the paper. TO finished the English editing. QZ contributed to the conception of the work and revised the paper.

### Conflict of interest statement

The authors declare that the research was conducted in the absence of any commercial or financial relationships that could be construed as a potential conflict of interest.
